# Whispers of Memory: How families navigate Dementia support beyond the Biomedical Lens in Nigeria

**DOI:** 10.1002/alz70858_096771

**Published:** 2025-12-24

**Authors:** Oluwagbemiga Oyinlola, Sussman Tamara, Magnus Mfoafo‐M’Carthy, Lawrence A Adebusoye

**Affiliations:** ^1^ McGill University, Montreal, QC, Canada; ^2^ McGill University, Montreal, ON, Canada; ^3^ Wilfrid Laurier University, Kitchner, ON, Canada; ^4^ Chief Tony Anenih Geriatric Centre, University College Hospital, Ibadan, Ibadan, Oyo State, Nigeria

## Abstract

**Background:**

Research on the experiences of caring for persons with dementia in African regions has tended to focus on caregivers' perceptions and experiences of using biomedical services and supports such as hospital centres. While valuable, this focus provides an incomplete picture, neglecting the broader network of support that caregivers rely upon. This study aims to investigate how all systems of support accessed by Yoruba caregivers in Nigeria work together to shape understanding of dementia and caregiving experiences.

**Method:**

This study used the interpretive phenomenology approach to understand the lived experiences of Yoruba families supporting persons with dementia (*n* = 15). Semi‐structured interviews (*family dialogue*) were conducted in the houses of these families in the presence of persons with dementia. All interviews were audio‐recorded, transcribed, translated, and reflexively analyzed using emergent themes.

**Result:**

While urban families (*n* = 11) benefited from structured hospital care (*n* = 10), rural families (4) relied heavily on traditional/faith‐based healers, neighbours, landlord associations and community elders. Families tended to seek help only in the later stages of dementia when behaviours had become more challenging. The types of services accessed appeared to be influenced by perceptions of dementia, geographical location (rural versus urban) and the advice given by trusted others. No matter where families sought assistance, they tended to feel supported when they received practical tips and guidance on how to best support their relatives with dementia. Those accessing traditional or faith‐based healing reported the additional benefits of spiritual guidance, hope, and a sense of community belonging.

**Conclusion:**

Our findings suggest the importance of improving dementia awareness amongst Yoruba caregivers in Nigeria so that help can be accessed sooner. It further suggests that improving caregivers' access to a variety of faith‐based traditional and medical supports may go a long way in improving caregivers' experiences in supporting persons with dementia.

